# In Vivo Indirect Insulin Dose Evaluation of Noninvasive Ultrasound-Mediated Transdermal Delivery Compared to Subcutaneous Injection

**DOI:** 10.3390/biomedicines14040900

**Published:** 2026-04-15

**Authors:** Osama Al-Bataineh, Rula Abdallat, Ausilah Alfraihat

**Affiliations:** Department of Biomedical Engineering, Faculty of Engineering, The Hashemite University, Zarqa 13133, Jordan; rulag@hu.edu.jo (R.A.); ausilaha@hu.edu.jo (A.A.)

**Keywords:** noninvasive transdermal insulin delivery, indirect functional insulin dose comparison, transdermal in vivo experiments, ultrasound-induced transdermal delivery

## Abstract

**Background**: Noninvasive transdermal insulin delivery using ultrasound technology has gained attention for improving the glycemic control of insulin-dependent patients. **Methods**: Indirect functional comparison and evaluation of insulin dosage, between noninvasive ultrasound-mediated transdermal delivery and needle injection methods, was achieved utilizing in vivo blood glucose measurements of temporary hyperglycemic rabbits. Nine rabbits were divided into three groups: (i) untreated control, (ii) subcutaneous injection and (iii) ultrasound-mediated transdermal delivery. Animals were anesthetized using a combination of ketamine hydrochloride and sodium xylazine to produce temporary hyperglycemic rabbits during the experiments. The rabbits in the control group did not receive insulin, while the animals in the ultrasound group received insulin transdermally for 10 min utilizing a customized single-element piston-shaped ultrasound transducer operated by multi-frequency electrical signals from 100 to 200 kHz. Rabbits in the direct subcutaneous injection group were anesthetized and injected with 0.25 units/kg of insulin. **Results**: With an initial blood glucose baseline level of 228.7 ± 13.1 (mg/dL) for all rabbits, the in vivo results of control group showed an increase above the baseline by 129.7 ± 27.3 (mg/dL) at the end of the in vivo experimental period (80 min). However, the ultrasound-mediated delivery and subcutaneous injection groups showed noticeable statistically significant percentage reductions in blood glucose levels by 43.9 ± 5.4 and 42.7 ± 6.6, respectively, compared to the control group by the end of the in vivo experiments. **Conclusions**: In vivo glucose response results confirmed that piston-shaped ultrasound transducers achieved indirectly similar insulin dosage delivery by ultrasound energy for tested animals with no statistically significant differences once compared to the results of the subcutaneous needle injection group.

## 1. Introduction

Insulin therapy is the treatment of choice to regulate blood glucose levels of type-1 diabetes (T1D) and advanced type-2 diabetes (T2D) [1,2,3]. Subcutaneous insulin injection has been the standard therapy for diabetes management utilizing invasive hypodermic multiple daily injections (MDI) or pump-mediated infusion [1,2]. MDI of insulin may increase the risk of infection and reduce patients’ adherence due to enduring pain and needle phobia [2,3]. To overcome these problems, researchers had investigated transdermal insulin delivery as an appealing substitute to subcutaneous approaches for diabetes management. Transdermal insulin delivery systems were proposed to avoid insulin destruction, offer accurate release mechanisms utilizing noninvasive or minimally invasive setups, increase patients’ adherence, and decrease the risk of infection for enhanced glycemic control [2,3]. Inefficient passive insulin permeation through the skin due to the large molecular weight of insulin is still the main challenge for effective transdermal insulin delivery. To overcome this major challenge, many transdermal insulin delivery techniques were proposed, including: chemically enhanced [4], electrically stimulated [5,6], mechanical force-assisted [2,7], microneedle technologies [6,8], insulin nanoparticles [9], micro-emulsion for transdermal delivery of insulin [10], and vesicular systems [11]. Table 1 shows the proposed transdermal insulin delivery technologies with their common benefits and restrictions.

Transdermal insulin delivery utilizes conventional chemical enhancer molecules and membrane-permeable peptides. Chemical enhancers disrupt skin barriers and provide higher transport efficiency of insulin through the lipid bilayers in the stratum corneum of the skin [4]. This technology had limited efficiency for insulin delivery, due to a lack of controlled insulin dosage, possible insulin degradation, and prospective skin irritation [2,3]. Electrically stimulated transdermal delivery of insulin, on the other hand, employs high-voltage electrical pulses (Electroporation) or continuous application of low-level currents (Iontophoresis). These techniques provide additional forces by introducing transient perturbation of the stratum corneum to facilitate transdermal insulin delivery [5,6]. Electrically facilitated transdermal delivery of insulin suffers from robust controlled insulin release, cell damage or rupture after membrane discharge, and time consumption specifically for iontophoresis mechanisms [2,3]. Furthermore, microneedle transdermal insulin delivery technology causes painless disruption of the stratum corneum to reach both the epidermal and dermal layers for insulin release. Micro-scaled needles’ designs included: solid, hollow, coated, dissolving, degradable, and bio-responsive microneedle technologies [6,8]. Iontophoresis and electroporation electrically enhanced technologies were also combined with microneedles’ designs to advance transdermal insulin delivery to improve diabetes management [6]. Microneedle techniques suffer from potential breakage inside tissue layers, toxicity concerns of microneedles’ materials, lower dosage precision, and possible skin irritation or infection [2,3]. Drug carriers and insulin vehicles were also utilized including vesicular formulations and micro-emulsion techniques [10,11]. These techniques suffered from insulin leakage and lower stability for transdermal insulin delivery [3]. Moreover, nanoparticle technology was investigated for sustained drug release of uniform spherical insulin nanoparticles that permeate efficiently across the skin utilizing supercritical anti-solvent micronization procedures. This technique required specialized and expensive equipment for proper usage [3,9]. Furthermore, mechanical-force-triggered insulin delivery technology employed ultrasound (US) waves and jet injection techniques [7] to accomplish transdermal glycemic management. Jet injection applied a high-speed narrow stream containing insulin to create tiny holes through the skin to dispense the insulin solution through the skin layers. Jet injection may cause bruising, bleeding, and pain due to high-pressure spray [2,3]. Ultrasound waves were hypothesized to enhance the transdermal permeability of insulin by the effects of generated interstitial microbubbles [2,3]. In fact, interstitial microbubble cavitation occurs due to the nucleation of slight gaseous voids during the negative pressure cycle of the ultrasound wave, trailed by the growth of microbubbles during consequent pressure cycles [12]. The cavitation threshold (lowest ultrasound intensity vital for the inception of cavitation) was inversely related to US driving frequencies and directly related to driving pulse length [12,13].

Ultrasound technology has gained attention as a potential noninvasive transdermal insulin delivery technique for improving the glycemic control of insulin-dependent diabetic patients [2,3,14]. This technology has been found to actively enhance transdermal drug delivery in a noninvasive manner referred to as sonophoresis [15]. Unlike traditional invasive needle injection therapy, US technology is anticipated to manage glycemic conditions of T1D and advanced T2D patients utilizing noninvasive US energy with increased adherence, reduced infection, and less probable damage to peripheral nerves [2,3,14]. Moreover, this noninvasive technology may help glycemic patients to get rid of the necessity for daily needle injections and pain and suffering [2,3]. US energy is thought to increase skin permeability due to the generation of interstitial microbubble cavities [13,16,17,18,19,20]. In fact, numerous mechanisms, in addition to interstitial microbubble cavitation, were suggested to explain the skin’s permeability increase due to US exposure, including temperature elevation, initiation of convective passage and mechanical effects. However, trial outcomes showed that cavitation effects were the principal contrivance in improving trans-skin drug delivery [20,21]. Moreover, researchers assured us that cavitation effects exterior to the skin had irrelevant effects on sonophoresis compared to interstitial cavitation underneath skin layers [12,20,21]. Studies have also concluded that it is difficult to generate cavitation at higher operating frequencies (>2.5 MHz) due to the short duration of oscillating acoustic negative pressures that reduce diffusion into cavitation nuclei [20,22,23]. For in vivo sonophoresis experiments, US transducers are considered as remote interstitial microbubble generators within targeted living tissues due to the existence of interstitially dissolved gases [13]. Moreover, the growth of variable oscillatory volumes of interstitial microbubbles in living tissues may act as micro-machines to transform radiated energy into a form of fluctuations and perturbations to temporarily increase skin permeability and porosity [14]. These hypothesized, developed microbubbles transform radiated mechanical energy within interstitial tissues and induce a dragging mechanism on drug molecules transdermally toward the circulation system. Depending on electrical driving and the control parameters of US transducers, such as driving frequencies, input power, exposure periods, and tuning circuitry, interstitial microbubbles could be initiated in either transient or stable forms. Transient microbubbles endure growth to a definite volume then collapse, resulting in shock waves and high microscopic temperatures. The oscillation of microbubbles near the lipid bilayer interfaces of the living cells and the shock waves from their collapse may lead to structural disordering that enhances the permeation of drugs through the stratum corneum [20,24,25,26]. Stable interstitial microbubbles, on the other hand, may possibly produce pulsating effects with microstreaming and shear forces affecting surrounding cells’ membrane structures and ultimately skin permeability [13,15,20]. Microbubble technologies, on the other hand, have been used in many engineering fields and applications including US radiography contrast agents [27], therapeutic applications [27], environmental pollution control [28], and numerous applications in chemical technologies [29].

Acoustical transient microbubble cavitation effects have been well documented in various applications of chemical technologies, specifically in the synthesis of functional materials, emulsification, cleaning, and processing of chemical reactions, known as sonochemistry technology [29]. Sonochemistry utilizes bulky and tuned US transducers resonating at low frequencies between 20 and 40 kHz due to the effectiveness in chemical applications [29]. Following the steps of sonochemistry, many researchers managed to apply US transducers with resonance frequencies between 20 and 40 kHz for in vivo trans-skin noninvasive insulin delivery using multi-element (ME) cymbal transducers [29,30,31,32,33], commercially used sonicators (VCX 400) [34], and ME disk transducers [35]. Pulse mode at the tuned resonance frequency had been applied to US transducers for transdermal insulin delivery including in vivo rats [30,33,34,35], rabbits [31], and pigs [32]. Various mechanisms of increased skin permeability, besides interstitial microbubbles, were established from trans-skin insulin studies on in vivo hairless rats and in vitro human skin [20,36], including thermal effects and convective transport through hair follicles and sweat glands [20,37]. As explained earlier, in vivo and in vitro studies showed that acoustic interstitial microbubbles played a more noteworthy role in sonophoresis than thermal and convective transport mechanisms [12,20]. Since acoustic microbubble cavitation in interstitial tissues can also be generated above 40 kHz, a much broader range of frequencies has generally been tested for transdermal drug delivery (sonophoresis), starting from low frequencies of 20 kHz until 16 MHz [17,38,39]. For transdermal insulin delivery, single-element (SE) piston-shaped US transducers, operated utilizing multi-frequencies ranging from 100 kHz to 1 MHz, were tested to prove the concept of in vivo insulin delivery utilizing temporarily hyperglycemic mice [14,40] and rabbits [40,41,42]. These transducers were operated using frequency sweep mode that presumably covered their underwater resonance frequencies to achieve in vivo animal experiments. Table 2 shows in vivo animal’ experimental studies utilizing ultrasound technology for noninvasive transdermal insulin delivery.

The results of in vivo rat experiments employing single-frequency pulse-driven ME cymbal transducers [30,33,34] indicated an instant drop in blood glucose levels following the administration of insulin. Furthermore, in vivo rat trials verified that single-frequency pulse-driven ME disk-shaped transducers [35] were able to deliver insulin transdermally in a noninvasive way. On the other hand, in vivo results of multi-frequency sweep-driven SE piston-shaped transducers showed variable behaviors of glucose level reductions depending on driving frequencies 1 h post-exposure (5 min for mice [14,40] and 10 min for rabbits [40,41,42]). Improved delivery mechanisms were linked to low-frequency driving signals from 100 to 200 kHz due to comparatively higher fluctuating volumetric sizes of hypothesized generated interstitially oscillatory microbubbles [14,40]. Few studies compared insulin dosages between sonophoresis and direct subcutaneous injection methods utilizing in vivo rat models [33,35]. The results of dose comparisons between insulin sonophoresis across Hairless Albino Westar Rats’ skin and subcutaneous insulin injection showed that the reductions in glucose concentration compared to the control group were 68% and 52%, respectively, for the subcutaneous direct injection group and the 60 min pulse-driven exposure group [35]. Moreover, Eun-Joo et al. showed that blood glucose reductions in Sprague-Dawley Rats were 53% and 76% for the subcutaneous direct injection group and the 60 min pulse-driven exposure group, respectively [33].

**Figure 1:**
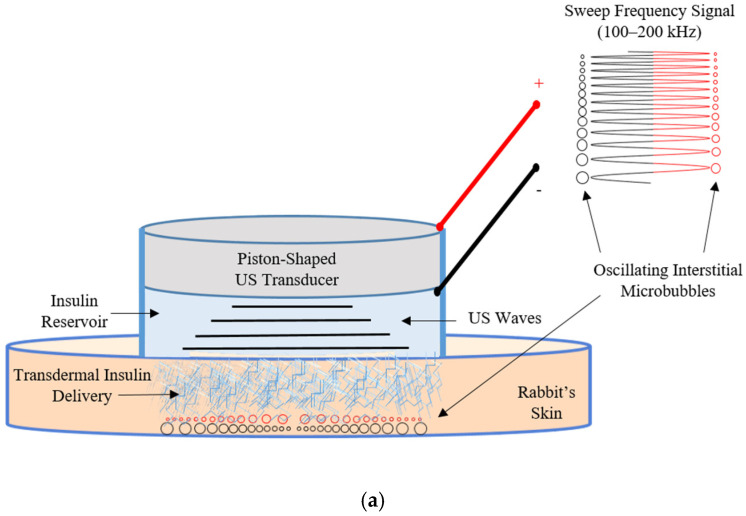

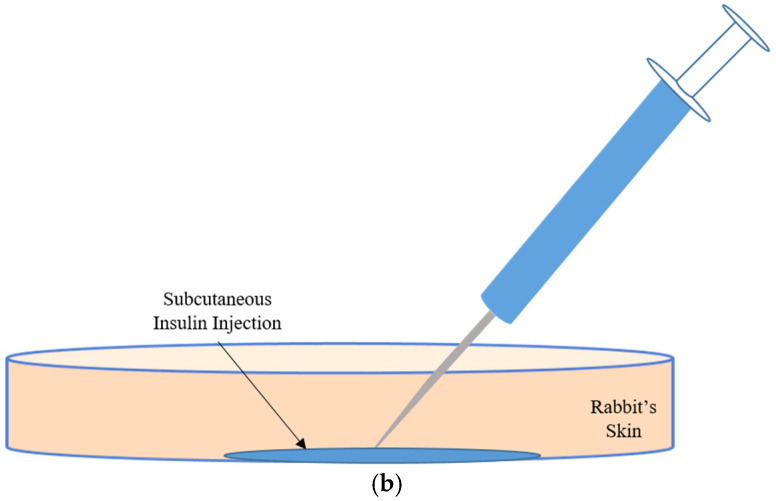
In vivo dose comparison between (**a**) US-mediated trans-skin insulin delivery using sweep frequency (100–200 kHz) excitation of piston-shaped US transducer and (**b**) traditional subcutaneous insulin needle injection.

Due to interdisciplinary approaches of insulin sonophoresis by US transducers utilizing interstitial microbubble generation and oscillation effects, engineering aspects are considered essential to initiate, control, and optimize temporary permeation of the targeted tight skin layers. These aspects bridge engineering technologies and biomedical sciences to optimize transdermal insulin delivery technology aimed at benefitting T1D and advanced T2D patients. To supplement the literature with more data regarding insulin dosages of sonophoresis utilizing multi-frequency excitations of US transducers [14,40], it is necessary to evaluate piston-shaped transducers and to compare their delivery results with traditional insulin injection techniques. Functional comparison of insulin dosage utilizing in vivo glucose level measurements between transdermal insulin delivery using piston-shaped transducers and direct insulin injection techniques may improve control capabilities over induced interstitial microbubbles and optimize insulin sonophoresis with enhanced driving parameters. In order to reveal essential control factors of the anticipated in vivo transdermal insulin delivery system, an engineering optimization of electrical driving parameters of US transducers can be inferred from dose comparison results with traditional direct insulin injection methods. Figure 1a shows the proposed US mechanism of insulin delivery across the tight skin layers utilizing oscillation effects of variable generated volume sizes of interstitial microbubbles. These assumed variable-sized microbubbles work as micro-machines to facilitate insulin permeation due to volume changes following negative and positive pressure cycles during frequency sweeping periods of the driving signal. As shown in Table 2, continuous sweeping of the driving frequency (from 100 to 200 kHz) during insulin delivery periods (5 min for rats and 10 min for rabbits) was capable of producing effective postulated interstitial microbubbles, leading to skin permeability increase. Reductions in insulin delivery periods were considered an appealing advancement for multi-frequency over single-frequency driving protocols of US transducers. The electrical driving parameters shown in Figure 1a, utilizing continuous application of a sweeping frequency signal from 100 to 200 kHz, satisfy the requirements of hypothesized generation and oscillation of interstitial microbubble cavities underneath targeted skin layers. Figure 1b shows that the invasive traditional needle injection method may cause needle phobia, low patient adherence, and elevated infection rates for T1D and advanced T2D patients. The purpose of this study was to evaluate and compare in vivo insulin dosages between noninvasive transdermal insulin delivery techniques (utilizing US transducers driven with multi-frequency signals from 100 to 200 kHz) and direct subcutaneous invasive injections of insulin depending on a functional comparison utilizing in vivo glucose level measurements of temporary hyperglycemic rabbits on the road to advance the engineering of a noninvasive robust transdermal insulin delivery system.

## 2. Materials and Methods

### 2.1. Ultrasound Transducers and Driving Setup

**Figure 2:**
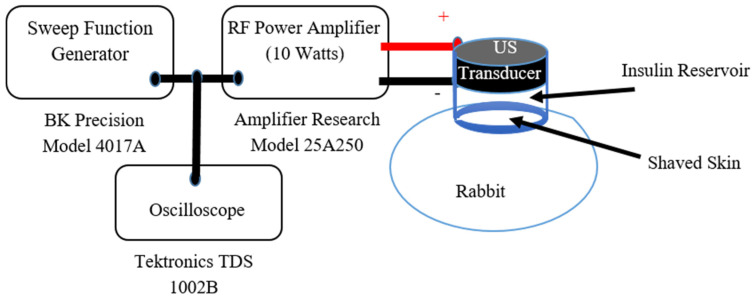
The driving setup of a customized piston-shaped transducer via power-amplified electrical signal utilizing sweep function generator and an RF power amplifier for in vivo noninvasive transdermal insulin delivery of an anesthetized local rabbit.

The driving parameters of the US transducers were carefully chosen to enhance skin permeability, while reducing insulin administration periods, utilizing continuous sweep multi-frequency modes of operation [14,40,41,42]. Piston-shaped Lead Zirconate Titanate type 4 (PZT4) transducers were obtained from Piezo Kinetics Incorporation (Piezo Kinetics, Inc., Bellefonte, PA, USA) with 30 mm diameters and 9.0 mm thicknesses. Table 3 summarizes essential parameters for in vivo animal experiments including physical properties and electrical driving conditions of piston-shaped transducers. Customized cylindrical insulin reservoirs with a volume capacity of around 2.0 mL were built for these transducers to hold insulin during in vivo transdermal insulin delivery experiments. The process of building insulin reservoirs to accommodate in vivo trials was described elsewhere [14,40,41,42]. Piston-shaped US transducers were excited using multi-frequency sweep mode of operation from 100 kHz to 200 kHz [14,41]. The frequencies of the driving electrical signals were continuously varied from a lower (F_Low_ = 100 kHz) to higher (F_High_ = 200 kHz) frequency range using a sweep function generator (BK Precision model 4017A, Yorba Linda, CA, USA) repeatedly every 10 s (sweep period (SP)) throughout the US exposure period (EP), which was set to 10 min for each in vivo insulin delivery experiment. As shown in Figure 2, a radio frequency (RF) power amplifier (Model 25A250, Amplifier Research, Souderton, PA, USA) with an input power of 10 Watts was set to drive piston-shaped PZT4 with frequency swept signals from the sweep function generator. The electrical driving signal from the sweep function generator was monitored using an oscilloscope (TDS 1002 B, Tektronix, Beaverton, OR, USA).

### 2.2. In Vivo Animal Experiments

**Figure 3:**
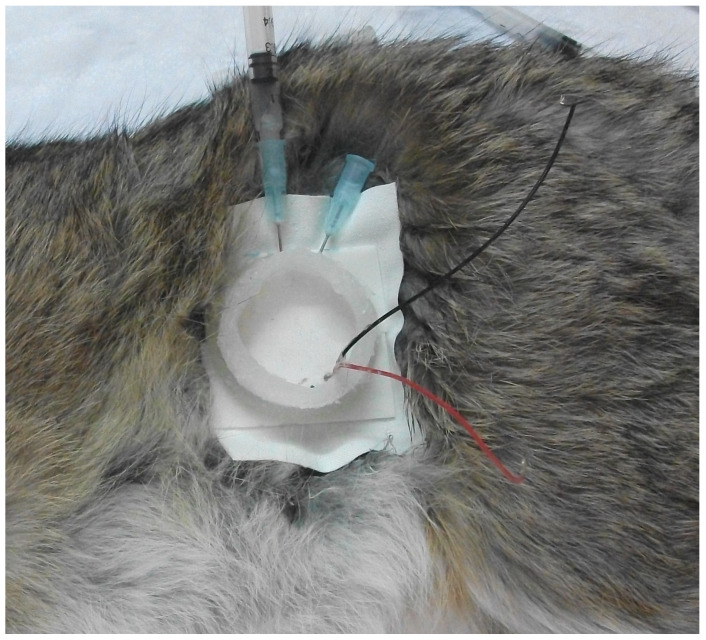
Photograph of an anesthetized rabbit during preparation of the insulin sonophoresis delivery phase.

Nine domestic rabbits (1.5–2.5 kg), including 5 males and 4 females, were obtained from the local market and housed in the animal housing facility of the Hashemite University. Animals were closely monitored and fed twice a day with access to drinking water. To achieve in vivo animal experiments, the animals were randomly distributed into three sets including a control group (COG, n = 3), an ultrasound-mediated transdermal insulin delivery group (USG, n = 3), and subcutaneous insulin injection group (SIG, n = 3). Direct measuremnets of blood glucose levels during in vivo experiments allowed for insulin dose comparison utilizing functional rather than quantitative measuremnts between the (USG) and (SIG) relative to the untreated control group (COG). The animal care committee of the Hashemite University approved the anesthesia, treatment procedures and protocols for in vivo local rabbit trials of this research numbered “H-1/11/2008”. Each animal was intramuscularly anesthetized (Figure 3) with a mixture of ketamine hydrochloride (60 mg/kg, TEKAM 50 mg/mL, HIKMA Co., Amman, Jordan) and sodium xylazine (10 mg/kg, XYLA-JECT 20 mg/mL, Adwia Co. S.A.E., 10th Ramdan City, Egypt). This mixture of ketamine/xylazine is known to momentarily cause hyperglycemic conditions during anesthesia of targeted animals [43]. Anesthetized local rabbits were laid on a heated table to control and maintain their body temperature at 37 °C during the experimental periods. The abdomen areas of the anesthetized rabbits were gently shaved using an electric shaver, and a depilatory agent (Nair^®^, Church & Dwight Co. Inc., Ewing Township, NJ, USA) was applied to the skin of the animals (USG and SIG) to eliminate any remaining hair. A hollowed medical plaster was bonded over the shaved area with double-faced foam tape, which enabled US transducers to be fixed in place with their insulin reservoirs close to the rabbits’ shaved skin. Two 16-gauge (G) needles were inserted via the silicone housing between the face of the transducer and the shaved skin. The reservoir was filled with 2 mL of insulin (Mixtard^®^ 30, Novo Nordisk, Bagsværd, Denmark) using a syringe attached to one of the pre-attached needles. A 30% fast-acting biphasic insulin type (Mixtard^®^ 30) was chosen to prevent hypoglycemic conditions for anesthetized rabbits during short investigational phases (80 min) from the start of US exposure or insulin administration periods (10 min). Trapped air inside insulin reservoirs was allowed to escape during filling phases through the other pre-hooked needle. Preparation periods of the anesthetized rabbits were limited to less than 15 min until the start of the insulin sonophoresis experimentations. Insulin was held for 10 min, between the US transducer and the shaved skin of the tested USG rabbits, during US exposure periods. Animals in the subcutaneous injection of insulin group (SIG) were subcutaneously injected with 0.25 unit/kg insulin (Mixtard^®^ 30, Novo Nordisk, Bagsværd, Denmark) after the anesthesia process. Usually, the starting dose to treat diabetic animals is recommend to be 0.25–0.5 U/kg every 12 h [44]. For the control group (COG), however, only the anesthesia mixture was administered to the tested animals. At the beginning of the experiments, blood samples (0.3 mL) were collected from the ear vein of each rabbit for the baseline glucose level analysis at the zero time point. The blood glucose level (mg/dL) was determined using a Gluco-Track^®^ (Teco Diagnostics, Anaheim, CA, USA) blood glucose monitoring system. Insulin delivery to assigned animals of USG and SIG started immediately after the zero time point, with many blood samples (2–4 samples each time) collected every 10 min during the investigational periods (80 min). Furthermore, inspections of the targeted shaved skin of each rabbit were performed after the end of US exposure experiments to look for observable changes to the skin surface. Visual examination of ultrasound-exposed skin surfaces did not indicate any evident damage or alterations in the skin of all US-exposed rabbits. Following in vivo animal experiments, all rabbits were transferred back to the animal housing facility and were humanly treated according to the animal care committee’s approved regulations.

## 3. Results

**Figure 4:**
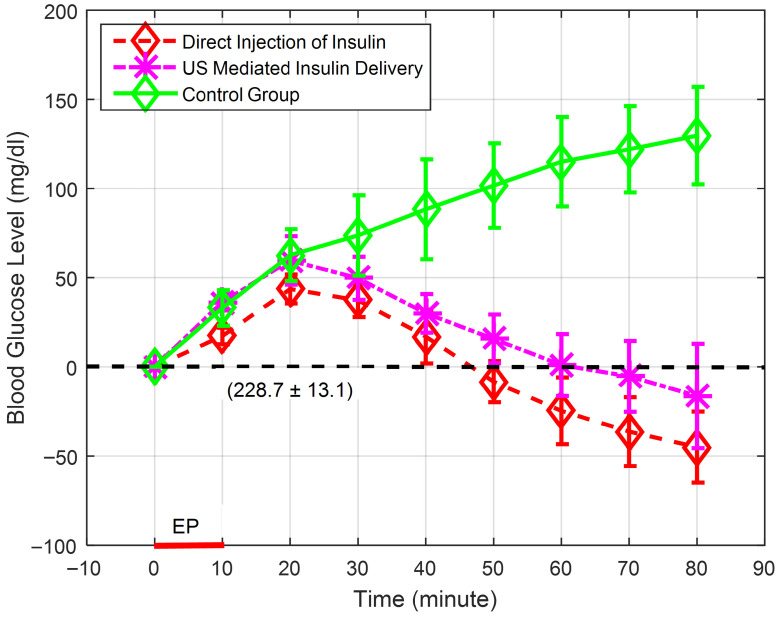
Relative blood glucose concentrations for the temporarily diabetic rabbit groups showing control group (COG (n = 3)), US-mediated insulin delivery group (USG (n = 3) and direct subcutaneous insulin injection group (SIG (n = 3)). The baseline (228.7 ± 13.1 mg/dL) and exposure period (EP (10 min)) are clearly shown.

The results of ultrasound-mediated transdermal insulin delivery in temporary diabetic local rabbits for the assigned groups (COG, SIG, and USG) are graphed (Figure 4) as the relative blood glucose level during the 80 min experiment’s period in terms of the mean and standard deviation. Table 4 shows the actual blood glucose levels (mg/dL) of the assigned groups during the experimental periods in terms of mean and the 95% confidence level results. A common baseline for initial values of glucose levels of all tested rabbits was assigned to eliminate differences between the starting points of the experimental groups (COG, SIG and USG) by subtracting the actual zero-time glucose level values of each tested rabbit from the rest of actual glucose level time points. Consequently, Figure 4 shows the definite glucose level, in terms of the mean and standard deviation, of the baseline (228.7 ± 13.1 (mg/dL), n = 9) and relative glucose levels for the assigned groups, with a zero-glucose level for the zero-time points. For the control group (COG), the relative glucose level increased to 129.7 ± 27.3 mg/dL in an eighty-minute period compared to the initial zero-time point. In contrast, the ultrasound exposure group (USG) showed an increase in blood glucose levels after ultrasound-mediated administration of insulin, with a peak value at the 20 min time point. Mixtard^®^ 30 insulin was designed to lower blood sugar within 30 min of administration. The peak value shown for the USG follows this behavior, as mentioned in the instruction sheet. After this peak value, the relative blood glucose levels sharply reduced to −16.3 ± 29.3 mg/dL by the end of the experimental period (80 min from the start of ultrasound-mediated insulin delivery time point). As explained for the USG, the relative glucose level values of the animals in the direct subcutaneous insulin injection group (SIG) showed a delayed response due to the design of Mixtard^®^ 30 insulin with a peak value at the 20 min time point, after which the relative blood glucose levels started to decrease, reaching −45.0 ± 19.9 mg/dL by the end of the experimental period. For ultrasound-mediated transdermal insulin delivery experiments (USG), the insulin delivery period (or US exposure period (EP)) was limited to 10 min from the allocated zero-time points.

Analysis of variance (ANOVA) was applied and followed by pair-wise post hoc multiple comparison tests utilizing MATLAB^®^ (R2015a) software (The MathWorks Inc., Natick, MA, USA) to determine the statistical significance between the actual blood glucose levels from the animals of the control group (COG, n = 3), ultrasound-mediated transdermal insulin delivery group (USG, n = 3), and subcutaneous insulin injection group (SIG, n = 3). Table 4 shows statistically significant differences between the groups, with a *p*-value ≤ 0.05 for all recorded time points of in vivo actual blood glucose levels for all assigned groups (COG, USG, and SIG). The interpretation of results utilizing a small sample size (n = 3 per group) is challenging to produce firm conclusions compared to large sample size trials with narrow confidence errors [45]. The confidence interval analysis results are shown in Table 4 for all recorded time points utilizing 95% confidence with a t-point of 4.3. Accordingly, the upper confidence interval values of the USG and SIG for the 40.0 and 50.0 min time points showed some overlap with the lower level of the confidence interval values of the COG, which reduces confidence in the significance claims for these time points. For this reason, the 40.0 and 50.0 time points were excluded from the statistically significant values. However, the analysis showed acceptable confidence errors for the 60.0, 70.0, and 80.0 min time points with no overlap between the confidence intervals of the USG and SIG with confidence intervals of COG. On the other hand, there were no significant differences between all groups for the first 30 min, indicating similar hyperglycemic behavior for all temporarily diabetic animals within all allocated groups. Consequently, the behavior of the USG and SIG showed statistically different behavior, with a *p*-value of ≤ 0.05 compared to the COG starting from the 60 min time points until the end of trial due to effective insulin delivery to the circulation system. Moreover, the ultrasound-mediated transdermal insulin delivery group (USG) and subcutaneous insulin injection group (SIG) showed no statistical differences between them during all time points, indicating that comparable amounts of insulin were delivered to the circulation system of the tested animals of both groups. To further evaluate statistically significant points starting from the 60 min time points, Table 5 shows actual percentages of blood glucose reduction levels of the insulin delivery groups (USG and SIG) compared to the control group (COG) in terms of the mean and standard deviation. The USG and SIG showed reductions in blood glucose levels by 35.4 ± 5.7% and 35.1 ± 5.2% at the 60.0 min time point and these percentages gradually increased to 43.9 ± 5.4% and 42.7 ± 6.6%, respectively, by the end of the in vivo experiments.

## 4. Discussion

The blood glucose levels of the ultrasound-mediated transdermal insulin delivery group (USG) and subcutaneous insulin injection group (SIG) showed no statistical significant differences, indicating the delivery of comparable amounts of insulin to the circulation systems of temporarily hyperglycemic assigned rabbits. However, once compared to the results of the control group (COG), both the USG and SIG showed statistically different behavior with actual reductions in blood glucose levels of 43.9 ± 5.4% and 42.7 ± 6.6%, respectively, by the end of the experiment’s duration. Ultrasound technology was found feasible to enhance the permeability and porosity of rabbits’ skin and noninvasively facilitated insulin passage across its tight layers. Table 6 shows an assessment of the results of in vivo insulin functional dose comparison trials utilizing a multi-element disk (MED) and multi-element cymbal (MEC) [33,35] with our single-element piston-shaped transducer for in vivo rabbit experiments. Jabbari et al. [35] and Eun-Joo et al. [33] reported noninvasive insulin delivery utilizing MED and MEC transducers to hairless albino Wistar (HAW) rats [33] and Sprague-Dawley (SD) rats [35] with pulsed excitation of sixty minutes to accomplish in vivo insulin delivery experiments. Although the conditions of in vivo experiments were varied among the tabulated trials, the ultrasound excitation period was reduced to 10 min for local rabbit trials compared to 60 min for HAW and SD rat trials [33,35]. This difference in US excitation periods is mainly due to variations in the design and driving mechanisms of in vivo experiments and US excitation modes with either pulsed single-frequency or continuous multi-frequency sweep excitation modes. Utilizing pulsed mode, researchers had driven MED and MEC US transducers at their tuned resonance frequencies for elongated insulin administration periods (60 min) to exploit radiated acoustic intensity interstitially to exposed tissues. Extended insulin administration periods may increase passive insulin penetration due to chemical effects of hair removal agents [14,44]. Due to passive permeation of the skin; indeed, hair removal chemical agents (Nair^®^, Church & Dwight Co. Inc., Ewing Township, NJ, USA) were testified to decrease glucose levels of chronic diabetic rats by 3%, 29%, and 43% after 30, 60, and 120 min, respectively, for 80 min insulin administration periods [46]. In fact, the contribution of passive insulin permeation of US-mediated insulin delivery results of this research is assumed to be insignificant due to the slow transport response through short periods of insulin administration or US exposure periods (10 min). However, this contribution is believed to be higher for US exposure or insulin administration periods greater than 30 min. It is evident that frequency sweep mode of operation reduced the period of insulin administration (US excitation—10 min) compared to pulsed mode excitations (60 min). Moreover, frequency sweep mode of operation is believed to excite a range of frequencies in an attempt to stimulate broader sizes of interstitial microbubble cavities to enhance the permeability and porosity of rabbits’ skin to ease and boost transdermal delivery of insulin molecules. The compromise of using this mode of operation was the lowering of insulin administration periods in favor of stimulating broader sizes of interstitial microbubble cavities to enhance the transdermal delivery of insulin. From an engineering point of view, US transducers utilizing sweep multi-frequency excitation generated more effective permeation microbubbles with reduced insulin administration periods compared to single-frequency operated transducers. The results of this study revealed that an SE piston-shaped transducer was capable of inducing an appropriate amount of interstitial microbubble cavities that allowed for a controlled dose of insulin to manage hyperglycemic conditions with only a 10 min excitation period. The tabulated results of actual percentages of blood glucose reductions in the insulin delivery groups compared to the control group in Table 6 shows that rabbits in the ultrasound-mediated insulin delivery group (USG) managed to indirectly deliver noticeable insulin dosages which were indicated by statistically significant results of both insulin delivery groups (SIG and USG). Additionally, the driving parameters of transducers of the USG during exposure periods suggested the dominance of stable cavitation formation over transient cavitation due to tuneless driving circuitry of US transducers [14,41]. The oscillation of stable cavitation microbubbles may contribute to microstreaming, microjets, fluid velocities, and shear forces on surrounding cells that momentarily alter cell membrane structure, function, and permeability or porosity [2,3]. For future clinical translations, a noninvasive robust insulin dosage delivery system utilizing sonophoresis technology still needs to be optimized due to some restrictions to this research study, including small size (n = 9) of in vivo tested animals, engagement of a temporary hyperglycemic animal model rather than diabetic animal model which may introduce confounding physiological responses, lack of long-term follow-up evaluation beyond the observation period (80 min), and the absence of exact measurements of acoustic intensities of US transducers and interstitial microbubble cavitations during in vivo experiments. In order to make a conclusion regarding clinical translation, future studies utilizing a chronic diabetic animal model should be designed with longer observation periods up to 12 h. Additional investigations, at the cellular and molecular levels, will be essential for the safety of the whole setup including quantitative measurement of insulin concentrations from blood samples [47] and histological or inflammatory assessment tests such as hematoxylin and eosin staining methods [48]. Histological or inflammatory assessment tests will be important to evaluate possible structural alterations of the epidermis and dermis including cellular disruption, edema, inflammation, or necrosis for the safety of the whole setup following recommendations and rules of the World Federation for Ultrasound in Medicine and Biology (WFUMB) [49] and the endorsements of the Advanced Technologies and Treatments for Diabetes Congress (ATTD) [1]. On the other hand, actual quantitative measurements of insulin concentrations along with glucose concentrations from blood samples during in vivo experiments will directly compare dose equivalency between insulin sonophoresis delivery and subcutaneous insulin injection methods for better pharmacokinetic profiles. In order to advance the suggested insulin delivery prototype toward a user-friendly tool, the challenge will be in reducing insulin administration periods (to less than 5 min [14]) while increasing insulin dosages to clinically advised ones up to 0.5 U/kg [44].

## 5. Conclusions

Single-element piston-shaped US transducers operating with sweep multi-frequencies from 100 to 200 kHz were found feasible for insulin delivery across rabbits’ skin layers in a noninvasive manner. In vivo experiments showed that multi-frequency operated piston-shaped transducers managed to functionally deliver noticeable insulin dosage of insulin to the tested animals and the behavior was statistically similar to the subcutaneous needle injection method. Continuous multi-frequency driving of US transducers improved the insulin delivery mechanism with reduced exposure and insulin administration periods. Sonophoresis of insulin in the form of a noninvasive patch is still under investigation due to the need for improved safety, efficiency, coziness, and further emphasis on engineering and ultrasound technologies.

## Figures and Tables

**Table 1:** Common restrictions of transdermal insulin delivery technologies.

Transdermal Insulin Delivery Technology	Benefits	Restrictions
Chemical Enhancers [4]	Patient satisfaction Inexpensive Flexibility and ease of use	Lack of controlled dosage Limited insulin delivery efficiency Skin irritation Possible insulin degradation
Electrically Aided [5,6]	Patient satisfaction Enhanced insulin permeation	Time consuming Potential cell damage or rupture Lack of controlled dosage
Microneedles [6,8]	Controllable insulin delivery rates Patient satisfaction	Potential breakage of needle Needle material toxicity Skin irritation and infection Lower dose precision
Mechanical Forces [2,7]	Less irritation Low infection risk	Potential bruising, bleeding, and pain due to high-pressure spray of jet injection Requirement of sophisticated driving devices Lack of controlled insulin dosage
Micro-Emulsion Vehicles [10]	Increased stability of insulin Versatile carrier	Leakage Time consuming Complex systems
Vesicular Systems [11]	Biodegradable Biocompatible Sustained insulin delivery	Leakage Expensive
Insulin Nanoparticles [9]	Low irritancy Sustained insulin release	Expensive Complicated techniques

**Table 2:** In vivo animal experiments showing US setup and in vivo animal models.

In Vivo Study	US Transducer	Exposure Period (min)	In vivo Animal Model
Boucaud et al. [34]	VCX 400 sonicator Pulsed Mode 20 kHz	15.0, 60.0	Hairless Rats
Lee et al. [31]	Multi-Element Cymbal Pulsed Mode 20 kHz	60.0	New Zealand White Rabbits
Smith et al. [30]	Multi-Element Cymbal Pulsed Mode 20 kHz	20.0, 60.0	Sprague-Dawley Rats
Park et al. [32]	Multi-Element Cymbal Pulsed Mode 20 kHz	60.0	Yorkshire Pigs
Jabbari et al. [35]	Multi-Element Disk Pulsed Mode 40 kHz	60.0	Hairless Albino Wistar Rats
Park et al. [33]	Multi-Element Cymbal Pulsed Mode 20 kHz	60.0	Sprague-Dawley Rats
Albataineh et al. [41]	Single-Element Piston Sweep Mode (100–1000) kHz	10.0	Local Rabbits
Albataineh et al. [42]	Single-Element Piston Sweep Mode (100–200) kHz	10.0	Local Rabbits
Albataineh et al. [40]	Single-Element Piston Sweep Mode (100–200) kHz	5.0, 10.0	Albino BALB/c Mice & Local Rabbits
Albataineh et al. [14]	Single-Element Piston Sweep Mode (100–650) kHz	5.0	Albino BALB/c Mice

**Table 3:** Piston-shaped transducer’s physical properties and electrical driving parameters of in vivo local rabbit trials.

Driving Frequency (kHz)	Piston-Shaped Thickness (mm)	In-Air Resonance Frequency (kHz)	Sweep Period (s)	Exposure Period (min)
100–200 kHz	9.0	225.8	10.0	10.0

**Table 4:** Actual blood glucose concentration levels of temporarily diabetic rabbits in terms of mean ± 95% confidence level (t-point = 4.3).

Actual Blood Glucose Concentrations of Temporarily Diabetic Rabbit Groups (mg/dL)			
Time Point (min)	Control Group COG (n = 3)	Transdermal Insulin Delivery Group USG (n = 3)	Direct Subcutaneous Insulin Injection Group SIG (n = 3))
0.0	224.7 ± 16.7	218.3 ± 22.2	243.1 ± 20.4
10.0	257.5 ± 25.4	254.6 ± 22.4	260.3 ± 31.4
20.0	287.3 ± 40.3	278.1 ± 17.2	286.6 ± 38.1
30.0	298.4 ± 58.5	268.2 ± 21.2	280.2 ± 31.1
40.0	313.1 ± 71.2	248.3 ± 9.5 *^,ol^	260.0 ± 22.2 *^,ol^
50.0	326.2 ± 64.0	234.0 ± 20.4 *^,nol^	234.7 ± 29.1 *^,ol^
60.0	339.6 ± 68.2	219.4 ± 22.4 *^,nol^	218.4 ± 31.9 *^,nol^
70.0	346.7 ± 65.2	213.1 ± 27.6 *^,nol^	206.7 ± 31.6 *^,nol^
80.0	354.3 ± 72.5	202.2 ± 50.3 *^,nol^	198.0 ± 36.6 *^,nol^

* Statistically significant values (*p* ≤ 0.05) compared to COG. ^ol^ Overlap with the 95% confidence intervals of COG. ^nol^ No overlap with the 95% confidence intervals of COG.

**Table 5:** Percentages of blood glucose reduction levels of the statistically significant time points of the actual in vivo results of the US-mediated transdermal insulin delivery group (USG) and subcutaneous insulin injection group (SIG) compared to the control group (COG).

Statistically Significant (*p* ≤ 0.05) Time Points (min)	Percentages of Actual Blood Glucose Reduction Levels Compared to Control Group (COG)	
	USG (%)	SIG (%)
60.0	35.4 ± 5.7	35.1 ± 5.2
70.0	40.2 ± 5.1	38.3 ± 5.0
80.0	43.9 ± 5.4	42.7 ± 6.6

**Table 6:** In vivo animal experiments showing percentages of blood glucose concentration in the exposure groups compared to the control group or baseline level.

In Vivo Study	US Transducer	Driving Frequency	In Vivo Model	Experimental Groups	GR% 60 min	GR% 80 min
Boucaud et al. [34]	VCX 400 SA = 3 cm^2^	UWRF = 20 kHz EG_20_	Hairless Rats (CG)	DI (IG)	70.0%	73.0%
				PM15 (EG_20_)	65.0%	66.0%
Jabbari et al. [35]	ME Disk SA = 4 cm^2^	IARF = 40 kHz EG_40_	HLAW Rats (CG)	DI (IG)	60.0%	65.0%
				PM60 (EG_40_)	38.0%	48.0%
Eun-Joo et al. [33]	ME Cymbal SA = 9 cm^2^	UWRF = 20 kHz EG_20_	SD Rats (No CG)	DI (IG)	38.0%	53.0%
				PM60 (EG_20_)	72.0%	74.0%
Current Study	SE Piston SA = 7 cm^2^	IARF = 226 kHz EG_100–200_	Local Rabbits (CG)	DI (IG)	35.0%	43.0%
				SM10 (EG_100–200_)	35.0%	44.0%

US: ultrasound, ME: multi-element, SA: approximate effetive surface area, SE: single-element, IARF: in-air resoance frequency, UWRF: underwater resonance frequency, EG_yy_: exposure group with ultrasound resonance frequency = yy, EG_100–200_: exposure group with sweeping frequency mode from 100 to 200 kHz, HLAW: hairless albino Wistar, SD: Sprague-Dawley, CG: control group, IG: injection group, DI: direct insulin subcutaneous injection (0.25 U/kg), PM60: pulsed mode for 60 min, SM10: sweeping mode for 10 min, GR% xx min: glucose reduction percentage at xx minutes from the beginning of experiments.

## Data Availability

Research data supporting this publication are available upon request.
